# NanoBRET binding assay for histamine H_2_ receptor ligands using live recombinant HEK293T cells

**DOI:** 10.1038/s41598-020-70332-3

**Published:** 2020-08-06

**Authors:** Lukas Grätz, Katharina Tropmann, Merlin Bresinsky, Christoph Müller, Günther Bernhardt, Steffen Pockes

**Affiliations:** Institute of Pharmacy, University of Regensburg, Universitätsstraße 31, 93053 Regensburg, Germany

**Keywords:** Biochemistry, Biological techniques, Chemistry

## Abstract

Fluorescence/luminescence-based techniques play an increasingly important role in the development of test systems for the characterization of future drug candidates, especially in terms of receptor binding in the field of G protein-coupled receptors (GPCRs). In this article, we present the establishment of a homogeneous live cell-based BRET binding assay for the histamine H_2_ receptor with different fluorescently labeled squaramide-type compounds synthesized in the course of this study. Py-1-labeled ligand **8** (UR-KAT478) was found to be most suitable in BRET saturation binding experiments with respect to receptor affinity (p*K*_d_ = 7.35) and signal intensity. Real-time kinetic experiments showed a full association of **8** within approximately 30 min and a slow dissociation of the ligand from the receptor. Investigation of reference compounds in BRET-based competition binding with **8** yielded p*K*_i_ values in agreement with radioligand binding data. This study shows that the BRET binding assay is a versatile test system for the characterization of putative new ligands at the histamine H_2_ receptor and represents a valuable fluorescence-based alternative to canonical binding assays.

## Introduction

**Figure 1:**
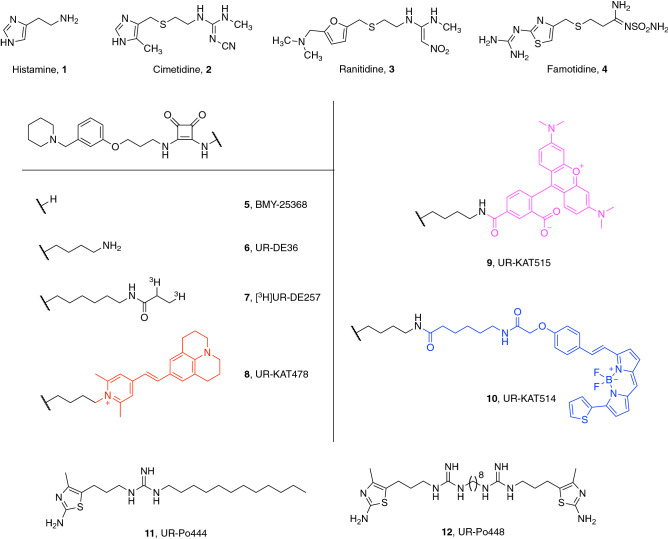
Structures of reported reference compounds (**1**–**7**, **11**–**12**) and the synthesized fluorescent ligands **8**–**10** for the histamine H_2_ receptor.

The histamine H_2_ receptor (H_2_R), which is activated endogenously by the biogenic amine histamine (**1**, Fig. 1), is a long known member of rhodopsin-like receptors (class A), the largest and best studied group of G protein-coupled receptors (GPCRs)^1–4^. It represents an established target for the treatment of gastroesophageal reflux disease (GERD) and peptic ulcer, with H_2_R antagonists, like cimetidine, ranitidine and famotidine (**2**–**4**, Fig. 1) being some of the first blockbuster drugs on the market in the 1970s^5^. Current research on CNS-penetrating H_2_R ligands, especially agonists, are ongoing, to get a better understanding of the role of the H_2_R in the brain, as little is known about that so far^6^. Since the H_2_ receptor has been described as being located in postsynaptic neurons and being involved in cognitive processes, it is discussed that stimulation of neuronal H_2_Rs could have similar positive effects on memory and learning as antagonizing the H_3_R^7–9^, which makes the H_2_R an interesting target for future drug development.

One of the first steps in developing novel ligands is the investigation of binding properties at the respective target. Until now, the characterization of potential ligands in terms of receptor binding is mostly done performing radioligand binding experiments. Despite its high sensitivity, the use of radiolabeled substances is usually connected with some drawbacks. In addition to the constantly increasing costs, the availability as well as the quality of commercial radioligands often declines. Furthermore, the management of radioactive waste is becoming increasingly regulated and expensive. To circumvent these issues, techniques using fluorescently labeled ligands like flow cytometry and the recently described BRET-based binding assay^10,11^, which has been adapted to several G-protein coupled receptors (GPCRs)^12–24^, have gained great importance.

For the NanoBRET binding assay the NanoLuc, a genetically engineered luciferase^25^, is fused to the N-terminus of the GPCR of interest^10^. After addition of the substrate the enzyme catalyzes an oxidation reaction, which is accompanied by the emission of blue light. Once a suitable fluorescent ligand binds to the tagged receptor, the ligand fluoresces due to bioluminescence resonance energy transfer (BRET). This transfer can only occur when the ligand is in close proximity to the bioluminescent donor, resulting in the observation of a lower non-specific binding as mainly the receptor-bound fraction of the fluorescent ligand is detected. Additionally, the ligand binding process can be followed in real time and not only after equilibrium is reached, which gives deeper insight into the kinetic behavior of the ligand.

In this study we established a BRET-binding assay for the histamine H_2_ receptor. Therefore we synthesized three differently labeled fluorescent ligands (**8**–**10**, Fig. 1), structurally derived from BMY-25368 (**5**, Fig. 1), a potent and long-acting histamine H_2_ receptor antagonist developed by Brystol-Myers in the 1980s^26^, and radioligand [^3^H]UR-DE257 (**7**, Fig. 1) from our laboratory^27,28^. These fluorescent tracers were tested for their suitability in the BRET binding assay.

## Results and discussion

### Synthesis of the fluorescent ligands

**Figure 2:**
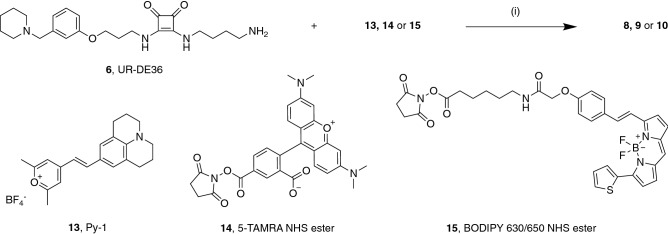
Synthesis of fluorescent ligands **8**–**10**. Reagents and conditions: (i) **6** (1.5 equiv.), NEt_3_ (7.5 or 11 equiv.), **13**, **14** or **15** (1 equiv.), DMF, rt, 2 h.

The synthesis of precursor UR-DE36 (**6**, Fig. 1) was carried out as previously reported in five steps^28,29^. Subsequently, **6** was treated with the respective labeling reagent (**13**–**15**, Fig. 2) in the presence of triethylamine resulting in **8**–**10**. Whereas **14** and **15** were commercially available, **13** was synthesized as described^30^. Except for **8**, aminolyses worked with good yields. Analytical characterization (^1^H-NMR, HPLC purity) of the fluorescent ligands **8**–**10** is shown in the Supplementary Figures S1–S6.

### Properties of synthesized ligands

**Figure 3:**
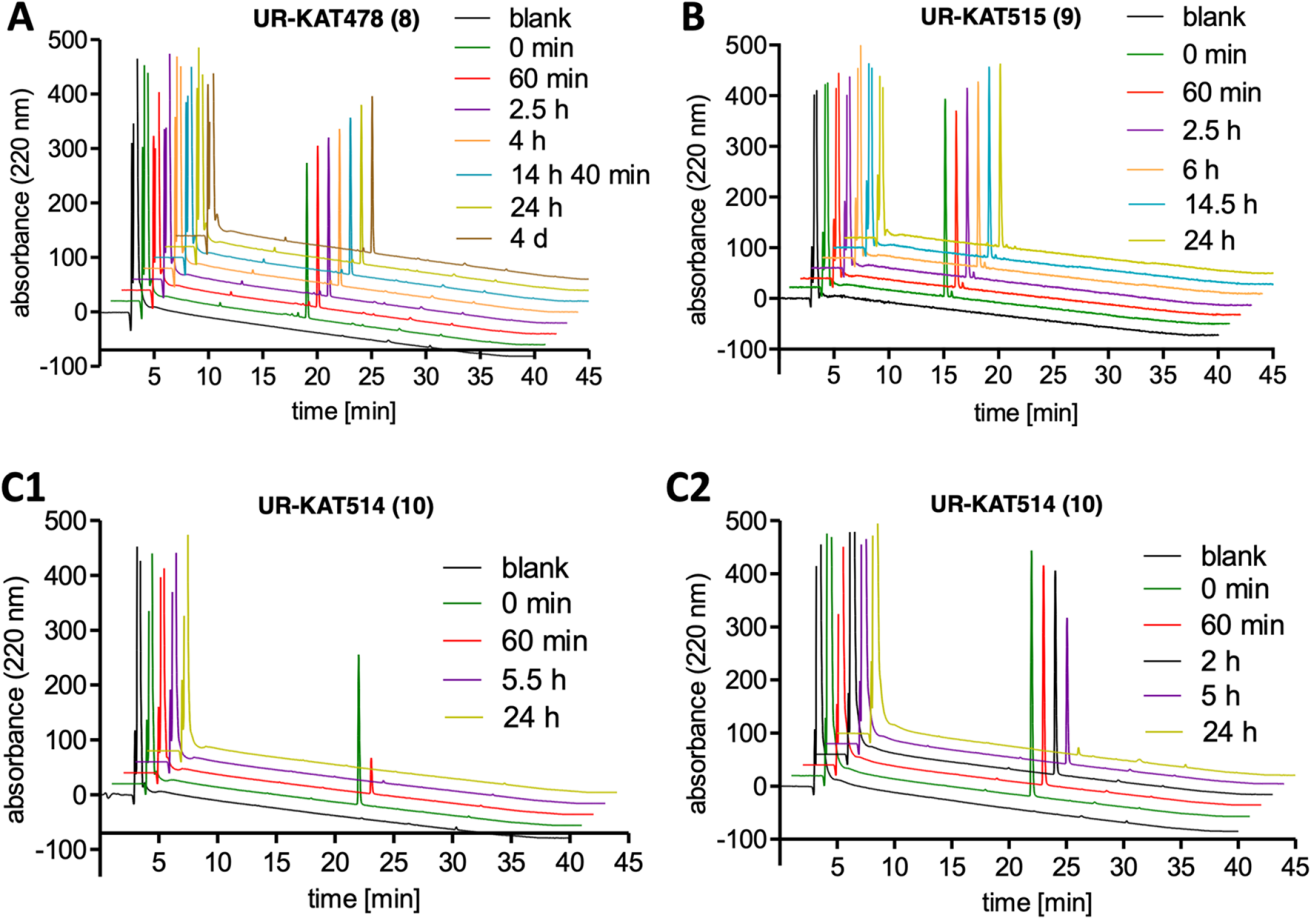
Chromatograms of **8** (**A**), **9** (**B**) and **10** (**C1** and **C2**) after different periods of incubation in binding buffer (pH 7.4, **A**, **B**, **C1**) or a mixture of binding buffer (pH 7.4)/DMSO 1:1 (**C2**) at rt.

The chemical stability of the fluorescent H_2_R ligands **8** (UR-KAT478), **9** (UR-KAT515) and **10** (UR-KAT514) was investigated under assay conditions (pH = 7.4) in binding buffer (BB; composition see Supplementary Information) at room temperature (rt) (Fig. 3, **A**,** B** and **C1**). Compounds **8** and **9** showed no decomposition during 96 h (Fig. 3, **A**) and 24 h (Fig. 3, **B**) incubation, respectively, and exhibited excellent chemical stability. The stability test with compound **10** also showed no chemical degradation. However, after only one hour almost no signal corresponding to compound **10** was detectable (Fig. 3, C1), most probably because of adsorption of the ligand to the plastic vessel. This phenomenon could be confirmed visually by staining of the vessel wall and discoloration of the analyte solution. Addition of DMSO to the binding buffer (BB/DMSO 1:1) reduced adsorption, which is depicted in Fig. 3, C2. In order to prevent the adsorption of the fluorescent ligands in the whole cell-based NanoBRET binding assay, 2% of bovine serum albumin (BSA) were added to the buffer used for all serial dilutions.

For a first pharmacological characterization of the synthesized compounds radioligand competition binding experiments, as well as flow cytometric saturation binding experiments were conducted. All ligands exhibited good affinities in the nanomolar range in both test systems, with **10** showing highest affinities (cf. Table 1; Supplementary Figs. S8, S9). Moreover, we investigated the ligands in a functional test system using a split-luciferase-based β-arrestin2 recruitment assay^31^. All ligands showed antagonistic behavior at the H_2_R (see Supplementary Figs. S10, S11) and the obtained p*K*_b_ values supported the findings from the binding assays described above (Table 1).

**Table 1:** Pharmacological characterization of the fluorescent ligands **8**–**10** at the hH_2_R in binding and functional assays.

Compound	NanoBRET^a^		RL comp. binding Sf9 membranes^b^		Flow cytometry^c^		β-arrestin2 recruitment^d^	
	p*K*_d_	N	p*K*_i_	N	p*K*_d_	N	p*K*_b_	N
**8**	7.35 ± 0.09	3	7.62 ± 0.06	3	7.13 ± 0.03	3	7.78 ± 0.15	6
**9**	6.84 ± 0.06	3	7.00 ± 0.10	4	6.25 ± 0.01	3	7.18 ± 0.13	5
**10**	8.59 ± 0.08	3	8.35 ± 0.05	3	7.86 ± 0.14	3	8.09 ± 0.04	3

Data represent mean values ± SEM from N independent experiments, as stated in Table 1, each performed in triplicate.

^a^NanoBRET binding experiments performed at live HEK293T cells stably expressing the NLuc-hH_2_R.

^b^Radioligand competition binding experiments with [^3^H]UR-DE257 (**7**) (hH_2_R, *K*_d_ = 11.2 nM, *c* = 20 nM, for representative radioligand saturation binding cf. Supplementary Fig. S7) on membrane preparations of Sf9 insect cells expressing the hH_2_R-Gsα_s_ fusion protein as described in the Supplementary Information.

^c^Flow cytometric measurements performed at HEK293T-hH_2_R-qs5-HA cells as described in the Supplementary Information.

^d^β-arrestin2 recruitment assays performed at HEK293T-ARRB2-H_2_R cells as described in the Supplementary Information.

### BRET-based binding assay at the H_2_ receptor

#### Saturation binding experiments

**Figure 4:**
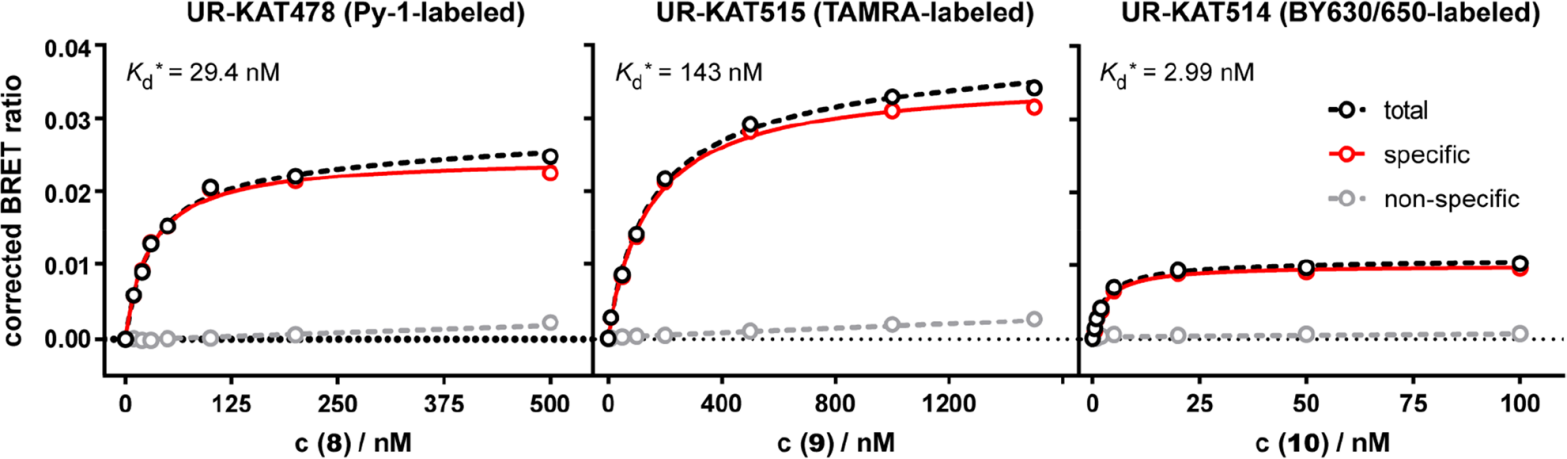
Representative isotherms from NanoBRET saturation binding experiments with **8**–**10** at the NLuc-hH_2_R, stably expressed in HEK293T cells. Non-specific binding was determined in presence of a 300-fold excess of famotidine. *: Indicated *K*_d_-values are results from single experiments. Error bars of total and non-specific binding represent SEM. The error of the specific binding was calculated according to the Gaussian law of error propagation. Each experiment was performed in triplicate (N = 3).

To investigate the suitability of the synthesized fluorescent ligands (**8**–**10**) for their use in the BRET-based binding assay, saturation binding experiments were performed at live HEK293T cells, stably expressing the NLuc-H_2_R fusion protein. As depicted in Fig. 4, binding was saturable for all compounds and equilibrium dissociation constants (*K*_d_/p*K*_d_ values, respectively) in the nanomolar range could be determined (cf. Table 1). In congruence with the results from the other binding assays, highest affinity was observed for the BODIPY 630/650 (BY630/650)-labeled ligand **10** with a p*K*_d_ of 8.59, followed by **8** (Py-1, p*K*_d_ = 7.35) and **9** (TAMRA) with a p*K*_d_ of 6.84 (Table 1).

However, beside the moderate signal-to-background ratio of **10,** a higher BRET ratio was found for **8** and **9**, which makes them more suitable as BRET acceptors (Fig. 4) for screening purposes. Due to its higher binding affinity, when compared to **9**, the Py-1-labeled fluorescent ligand **8** was used for further experiments.

#### Real-time kinetic experiments with **8**

**Figure 5:**
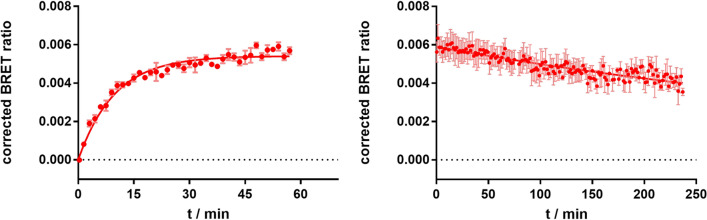
BRET-based specific binding kinetics of the fluorescent ligand **8** at the NLuc-hH_2_R, stably expressed in HEK293T cells. Left panel: Association of **8** (c = 50 nM) to the receptor; Right panel: Dissociation of **8** (c = 50 nM) after preincubation for 60 min. Dissociation was started by addition of famotidine (300-fold excess, c = 15 µM). Graphs show representative experiments (N = 4), each performed in triplicate. Error bars of specific binding represent propagated errors.

For the further characterization of **8,** real-time kinetic binding experiments were conducted (Fig. 5). Therefore, 50 nM of **8** were used to measure ligand association to the H_2_R (Fig. 5, left). The ligand was fully bound to the receptor after approximately 30 min. Dissociation of **8** was initiated by the addition of a 300-fold excess of famotidine (c = 15 µM) after preincubation (60 min) of the cells with fluorescent ligand (c = 50 nM; Fig. 5, right). Slow dissociation kinetics with a dissociation half-life of 300 min were observed and only a small amount of **8** was displaced within 240 min (35–40%). A similar behavior was also reported for the structurally related radioligand **7**, leading to the assumption that the pharmacological scaffold is responsible for this type of binding^28^. All kinetic parameters describing the binding of **8** are summarized in Table 2.

**Table 2:** Kinetic parameters of **8** at the NLuc-hH_2_R determined in the BRET-based binding assay.

	*k*_obs_^a^ (min^−1^)	*k*_off_^a^ (min^−1^)	*t*_1/2_^b^ (min)	*k*_on_^c^ (min^−1^ nM^−1^)	*K*_d(kin)_^d^ (nM)
8	0.093 ± 0.009	0.0023 ± 0.0002	300.4 ± 19.90	0.0018 ± 0.0002	1.30 ± 0.16

^a^Data represent mean values ± SEM from four independent experiments.

^b^Dissociation half-life *t*_1/2_ = ln(2)/*k*_off_*.*

^c^Association rate constant.

^d^*K*_d(kin)_ = *k*_off_/*k*_on_.

^b,c,d^Indicated errors were calculated according to the Gaussian law of error propagation.

#### Investigation of reported H_2_R ligands in BRET-based competition binding

**Figure 6:**
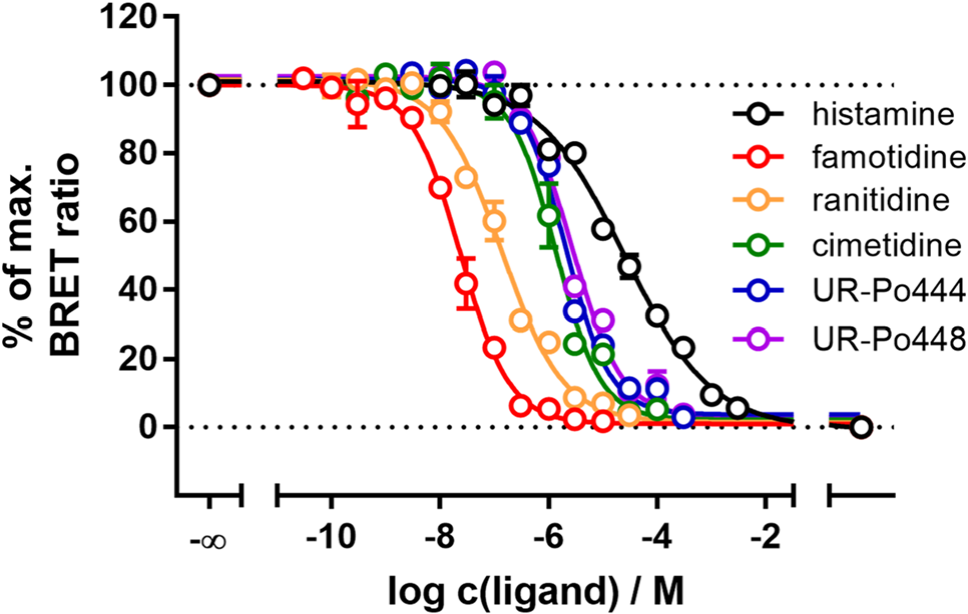
Displacement of the fluorescent ligand **8** (c = 50 nM) by reported H_2_ receptor ligands in BRET-based competition binding experiments at HEK293T cells, stably expressing the NLuc-hH_2_R. Data shown are means ± SEM from at least four independent experiments, each performed in triplicate.

To show the versatility of the presented assay principle, we additionally performed BRET-based competition binding experiments with different reported H_2_ receptor agonists and antagonists, using one fixed concentration of **8** (c = 50 nM) and various concentrations of the respective ligands (Fig. 6). Despite the slow dissociation kinetics of **8**, all ligands were able to totally displace the fluorescent tracer after 60 min. It is noteworthy that the displacement curve of histamine shows a markedly flatter slope (slope ± SEM = − 0.55 ± 0.03, N = 5) in comparison to the other tested competitive ligands, which could suggest the existence of a second receptor affinity state, as previously described^32^. However, this could not be clearly confirmed in a competition binding experiment using an extended set of concentrations (cf. Supplementary Fig. S12). Therefore, monophasic binding was assumed for all tested compounds. The p*K*_i_ values from the BRET competition binding assay are shown in comparison to radioligand binding data are shown in Table 3. Data reported for radioligand binding at CHO hH_2_R membranes^32^ are in good accordance with our NanoBRET data obtained at live recombinant HEK293T cells, while data acquired at Sf9 membranes expressing the hH_2_R-Gsα_s_ fusion protein^28^ show a larger deviation. It is conspicuous that agonists (**1**, **11**–**12**) show comparatively higher affinities at Sf9 membranes, whereas antagonists/inverse agonists (**2**–**4**) show lower affinities (cf. Table 3). A possible explanation for this observation could be the direct fusion of the receptor with the Gsα_s_, since the receptor is thereby permanently brought into an active receptor conformation favoring agonist binding. In contrast, antagonists and especially inverse agonists do not prefer this receptor state, which may lead to the observation of lower binding affinities. This is relevant as cimetidine (**2**), ranitidine (**3**) and famotidine (**4**) are also often described as inverse agonists at the hH_2_R, supporting our finding^33–35^. Another possibility for the evident discrepancy in the affinity for histamine (**1**) is the known allosteric effect of sodium on the binding of agonists to several GPCRs^36^. As the buffer used in radioligand binding assays at Sf9 membranes expressing the H_2_R is devoid of sodium ions, in contrast to the BRET assay and also to the binding experiments performed at CHO membranes^32^, we changed the assay procedure by adding sodium in a physiological concentration (c = 145 mM) to the binding buffer. This change resulted in a decrease in affinity for histamine (**1**) with a p*K*_i_ of 4.37 ± 0.02 (N = 3, cf. Table 3^#^) for histamine (**1**), which is now in good agreement to the binding constant from the BRET binding assay. Taken together the presented BRET-based approach yields comparable binding data for reported histamine H_2_ receptor ligands, which confirms the suitability of the test system in combination with the fluorescent ligand **8**.

**Table 3:** Binding data (p*K*_i_ values) of histamine (**1**), cimetidine (**2**), ranitidine (**3**), famotidine (**4**), UR-Po444 (**11**) and UR-Po448 (**12**) determined at the human H_2_R in the NanoBRET binding assay and radioligand competition binding assays.

Compound	NanoBRET^a^		RL comp. binding Sf9 membranes^b^		RL comp. binding CHO membranes^c^
	p*K*_i_	N	p*K*_i_	N	p*K*_i_
**1**	4.96 ± 0.09	5	6.27^28^; 4.37^#^	3	4.10; 5.69^d^^32^
**2**	6.30 ± 0.14	4	5.56 ± 0.14	3	6.18^32^
**3**	7.19 ± 0.09	4	5.76^28^	3	7.07^32^
**4**	7.94 ± 0.04	5	6.87^28^	3	7.80^32^
**11**	5.99 ± 0.09	4	6.60 ± 0.08	3	n.d
**12**	5.88 ± 0.08	4	6.34 ± 0.04	3	n.d

^a^Data represent mean values ± SEM from at least four independent experiments, each performed in triplicate. NanoBRET experiments were performed at live HEK293T cells stably expressing the NLuc-hH_2_R as described in Methods.

^b^Radioligand competition binding experiments were performed with [^3^H]UR-DE257 (hH_2_R, *K*_d_ = 11.2 nM, *c* = 20 nM, for representative radioligand saturation binding cf. Supplementary Fig. S7) on membrane preparations of Sf9 insect cells expressing the hH_2_R-Gsα_s_ fusion protein. ^#^Experiments were performed in analogy with ^b^, apart from the addition of 145 mM NaCl to the binding buffer.

^c^Data from radioligand competition binding experiments performed at CHO-hH_2_R membranes, expressing the hH_2_R with [^125^I]-iodoaminopotentidine^32^.

^d^Biphasic curve with high and low affinity state. Indicated p*K*_i_-values were re-calculated from published *K*_i_-values.

## Conclusion

In this study we report the development of a NanoBRET binding assay for the histamine H_2_ receptor including the synthesis and characterization of suitable fluorescent H_2_R ligands. As a homogeneous live cell-based assay, this assay allows for a convenient determination of affinity constants of putative H_2_ receptor ligands, independent of their mode of action without any washing or separation steps. The results from our BRET binding assay were well comparable to currently used radioactivity- or fluorescence-based (e.g. flow cytometry) binding assays. Furthermore, real-time kinetic measurements can be performed enabling a better resolved monitoring of ligand-receptor interactions. Prerequisite for the establishment of such assays is the availability of suitable fluorescent ligands. Therefore, we synthesized three differently labeled compounds, all of which have proven to be generally usable in BRET saturation binding experiments. Out of those, substance **8** turned out to be the best compromise with regard to receptor affinity and signal strength and was successfully used for further investigations. Until now, BRET binding assays have only been described for the histamine H_1,3,4_ receptors^22,23^, making this study close the gap of NanoBRET assays within the histamine receptor family. Thus, selectivity studies, which are essential for the development of new drug candidates, can be carried out using the same assay principle increasing the comparability of results. All in all, this study shows that the BRET binding assay is a valuable test system for the histamine H_2_ receptor and provides a novel fluorescence-based alternative to other conventional binding assays.

## Materials and methods

### Materials

Dulbecco’s modified Eagle’s medium (DMEM) and HEPES were purchased from Sigma-Aldrich (Munich, Germany). Leibovitz’ L-15 medium (L-15) was from Fisher Scientific (Nidderau, Germany). Fetal calf serum (FCS), geneticin and trypsin/EDTA (0.05%/0.02%) were from Biochrom (Berlin, Germany). Bovine serum albumin (BSA) was sourced from SERVA Electrophoresis (Heidelberg, Germany). Furimazine was from Promega, (Mannheim, Germany). Histamine dihydrochloride was from TCI Chemicals (Tokyo, Japan). Cimetidine was from Sigma-Aldrich (Munich, Germany). Famotidine and ranitidine hydrochloride were from Tocris Bioscience (Ellisville, MO, USA). All other chemicals and solvents were purchased from standard commercial suppliers [Merck (Darmstadt, Germany), Sigma-Aldrich (Munich, Germany), Acros Organics (Geel, Belgium), Alfa Aesar (Karlsruhe, Germany), abcr (Karlsruhe, Germany)] and were used as received. The fluorescent dyes BODIPY 630/650 X NHS ester and 5-TAMRA NHS ester were purchased from Lumiprobe (Hannover, Germany) or abcr (Karlsruhe, Germany) respectively, the pyrylium dye Py-1 was synthesized as previously published^30^. All solvents were of analytical grade.

### Synthesis and analytical data

#### General

NMR spectra were recorded on a Bruker Avance 600 (^1^H: 600 MHz) (Bruker, Karlsruhe, Germany) with deuterated solvents from Deutero (Kastellaun, Germany). HRMS was performed on an Agilent 6540 UHD Accurate-Mass Q-TOF LC/MS system (Agilent Technologies, Santa Clara, CA, USA) using an ESI source. Preparative HPLC was performed with a system from Waters (Milford, Massachusetts, USA) consisting of a 2524 binary gradient module, a 2489 detector, a prep inject injector, fraction collector III and the column was a Phenomenex Kinetex (250 × 21 mm, 5 µm) (Phenomenex, Aschaffenburg, Germany). As mobile phase, mixtures of MeCN and 0.1% aqueous TFA were used. UV detection was carried out at 220 nm. Freeze-drying was carried out using a ScanVac CoolSafe 4-15L freeze dryer from Labogene (LMS, Brigachtal, Germany), equipped with a RZ 6 rotary vane vacuum pump (Vacuubrand, Wertheim, Germany). Analytical HPLC experiments were performed on a 1,100 HPLC system from Agilent Technologies, equipped with Instant Pilot controller, a G1312A Bin Pump, a G1329A ALS autosampler, a G1379A vacuum degasser, a G1316A column compartment and a G1315B DAD detector. The column was a Phenomenex Kinetex XB-C18 column (250 × 4.6 mm, 5 µm) (Phenomenex, Aschaffenburg, Germany), tempered at 30 °C. As the mobile phase, mixtures of MeCN and 0.05% aqueous TFA were used. Gradient mode: MeCN/TFA (0.05%) (v/v) 0 min: 10:90, 30 min: 90:10, 33 min: 95:5, 40 min: 95:5, 43 min: 10:90, 50 min: 10:90; flow rate: 0.8 mL/min, t_0_ = 3.21 min; capacity factor k = (t_R _− t_0_)/t_0_. Absorbance was detected at 220 nm. Purity of the compounds was calculated as the percentage peak area of the analyzed compound by UV detection at 220 nm. The purities of the fluorescent ligands used for pharmacological investigation were ≥ 95%.

#### General procedure for the synthesis of the fluorescent ligands

The amine precursor UR-DE36 (**6**, (3-((4-Aminobutyl)amino)-4-((3-(3-(piperidin-1-ylmethyl)phenoxy)propyl)amino)cyclobut-3-ene-1,2-dione × 2 TFA), was synthesized as previously reported^28,29^.

Following labeling reactions were carried out in 1.5-mL Eppendorf reaction vessels. The amine UR-DE36 (**6**, 1.5 equiv.) was dissolved in 30 µL of DMF, before NEt_3_ (7.5 or 11 equiv.) was added. The labeling reagents (1 equiv.) were dissolved in 20 µL of DMF, added to the mixture and the vessel was rinsed twice with DMF (20 µL and 10 µL). The mixture was stirred at room temperature for 2 h. Subsequently, the reaction was stopped by addtion of 10% aqueous TFA (20 µL). The crude products were purified by preparative HPLC. The solvent was removed by lyophilization.

##### (E)-1-(4-((3,4-Dioxo-2-((3-(3-(piperidin-1-ylmethyl)phenoxy)propyl)amino)cyclobut-1-en-1-yl)amino)butyl)-2,6-dimethyl-4-(2-(2,3,6,7-tetrahydro-1H,5H-pyrido[3,2,1-ij]quinolin-9-yl)vinyl)pyridin-1-ium (**8**)^37^

The title compound was prepared from amine **6** (6.9 mg, 10.8 µmol), Py-1 (**13**, 2.8 mg, 7.2 µmol) and NEt_3_ (7.5 µL, 54 µmol) according to the general procedure yielding the product as a red solid (0.98 mg, 15%). RP-HPLC: 96.0% (t_R_ = 17.20 min, k = 4.36). ^1^H NMR (600 MHz, DMSO-*d*_6_) δ 9.22 (s, 1H), 7.77 (s, 2H), 7.68 (d, *J* = 15.9 Hz, 1H), 7.37 (t, *J* = 7.9 Hz, 1H), 7.10 (s, 2H), 7.07 (s, 1H), 7.05–7.00 (m, 2H), 6.88 (d, *J* = 16.0 Hz, 1H), 6.51 (s, 1H), 4.34–4.28 (m, 2H), 4.22 (d, *J* = 5.3 Hz, 2H), 4.05 (t, *J* = 6.0 Hz, 2H), 3.71–3.63 (m, 2H), 3.60–3.51 (m, 2H), 3.25 (t, *J* = 5.8 Hz, 4H), 2.85 (q, *J* = 10.9 Hz, 2H), 2.72 (s, 6H), 2.69 (t, *J* = 6.3 Hz, 4H), 2.03–1.96 (m, 2H), 1.86 (p, *J* = 6.1 Hz, 4H), 1.83–1.76 (m, 3H), 1.72–1.55 (m, 6H), 1.40–1.29 (m, 1H). HRMS (ESI–MS): *m/z* M^+^ calcd. for C_44_H_56_N_5_O_3_^+^: 702.4378; found: 702.4382; C_44_H_56_N_5_O_3_^+^ × C_4_HF_6_O_4_^-^ (930.00).

##### 2-(3,6-Bis(dimethylamino)xanthylium-9-yl)-5-((4-((3,4-dioxo-2-((3-(3-(piperidin-1-ylmethyl)phenoxy)propyl)amino)cyclo-but-1-en-1-yl)amino)butyl)carbamoyl)benzoate (**9**)

The title compound was prepared from amine **6** (5.8 mg, 9.0 µmol), 5-TAMRA NHS ester (**14**, 3.2 mg, 6.0 µmol) and NEt_3_ (9.3 µL, 67 µmol) according to the general procedure yielding the product as a pink solid (4.02 mg, 70%). RP-HPLC: 96.5% (t_R_ = 13.88 min, k = 3.32). ^1^H NMR (600 MHz, DMSO-*d*_6_) δ 8.35 (s, 1H), 8.91 (t, *J* = 5.2 Hz, 1H), 8.28 (d, *J* = 8.2 Hz, 1H), 8.66 (s, 1H), 7.79–7.44 (m, 2H), 7.38 (t, *J* = 7.9 Hz, 1H), 7.22–6.66 (m, 8H), 4.23 (s, 2H), 4.06 (t, *J* = 5.92 Hz, 2H), 3.69 (s, 2H), 3.60–3.53 (m, 2H), 3.38–3.34 (m, 2H), 3.33–3.12 (m, 14H), 2.92–2.80 (m, 2H), 2.01 (p, *J* = 6.3 Hz, 2H), 1.86–1.76 (m, 2H), 1.72–1.54 (m, 7H), 1.41–1.30 (m, 1H). HRMS (ESI): m/z [M + H]^+^ calcd. for C_48_H_55_N_6_O_7_^+^: 827.4127; found: 827.4126; C_48_H_54_N_6_O_7_ × C_2_HF_3_O_2_ (941.02).

##### (E)-6-(2-(4-(2-(5,5-Difluoro-8-(thiophen-2-yl)-5H-4λ^4^,5λ^4^-dipyrrolo[1,2-c:2′,1′-f][1,3,2]diazaborinin-3-yl)vinyl)phen-oxy)acetamido)-N-(4-((3,4-dioxo-2-((3-(3-(piperidin-1-ylmethyl)phenoxy)propyl)amino)cyclobut-1-en-1-yl)amino) butyl)hexanamide (**10**)

The title compound was prepared from amine **6** (4.2 mg, 6.5 µmol), BODIPY 630/650 X NHS ester (**15**, 2.9 mg, 4.3 µmol) and NEt_3_ (6.6 µL, 47 µmol) according to the general procedure yielding the product as a dark blue solid (3.25 mg, 69%). RP-HPLC: 98.3% (t_R_ = 20.84 min, k = 5.49). ^1^H NMR (600 MHz, DMSO-*d*_6_) δ 9.19 (s, 1H), 8.12 (t, *J* = 5.8 Hz, 1H), 8.03 (dd, *J* = 3.8, 1.1 Hz, 1H), 7.82 (dd, *J* = 5.0, 1.1 Hz, 1H), 7.78–7.70 (m, 2H), 7.62–7.57 (m, 3H), 7.41–7.34 (m, 3H), 7.30–7.25 (m, 3H), 7.08–7.04 (m, 3H), 7.04–6.99 (m, 2H), 6.94 (d, *J* = 4.2 Hz, 1H), 4.52 (s, 2H), 4.21 (d, *J* = 5.2 Hz, 2H), 4.04 (t, *J* = 6.1 Hz, 2H), 3.66 (s, 3H), 3.31–3.26 (m, 3H), 3.10 (q, *J* = 6.7 Hz, 2H), 3.02 (q, *J* = 6.6 Hz, 2H), 2.88–2.79 (m, 2H), 2.52–2.50 (m, 2H), 2.05–1.95 (m, 4H), 1.84–1.76 (m, 2H), 1.70–1.53 (m, 3H), 1.51–1.28 (m, 8H), 1.27–1.14 (m, 2H). HRMS (ESI): m/z [M + H]^+^ calcd. for C_52_H_61_BF_2_N_7_O_6_S^+^: 960.4460; found: 960.4471; C_52_H_60_BF_2_N_7_O_6_S × C_2_HF_3_O_2_ (1,073.99).

### Generation of plasmids

The cDNA coding for the human H_2_R was purchased from the Missouri cDNA resource centre (Rolla, MO, USA). The plasmid encoding NanoLuc was kindly provided by Promega (Mannheim, Germany). The sequences of the receptor and the luciferase were amplified using standard PCR techniques, introducing restriction sites at their respective 5′ and 3′ ends as well as the membrane signal peptide of the murine 5HT_3A_ receptor upstream of the luciferase gene. These were then cloned in-frame into the pcDNA3.1/myc-HIS (B) vector backbone separated by a flexible linker (-SGGGS-) to generate the plasmid encoding the NLuc-hH_2_R. All sequences were verified by sequencing (Eurofins Genomics, Ebersberg, Germany).

### Cell culture and transfection

All cells were routinely cultivated in DMEM + 10% FCS in a water-saturated atmosphere containing 5% CO_2_ at 37 °C and regularly monitored for mycoplasm infection using the Venor GeM Mycoplasma Detection Kit (Minerva Biolabs, Berlin, Germany). In order to generate stable transfectants, HEK293T wild-type cells were seeded at a density of 3 × 10^5^ cells/mL in a 6-well plate (Sarstedt, Nümbrecht, Germany) one day prior to transfection with 2 µg of cDNA using XtremeGene HP transfection reagent (Roche Diagnostics, Mannheim, Germany) according to the manufacturer’s protocol. After two days of incubation (water-saturated atmosphere, 5% CO_2_, 37 °C), transfected cells were trypsinized, transferred to a 15 cm cell culture dish (Sarstedt, Nümbrecht, Germany) in DMEM and geneticin was added at a final concentration of 1 mg/mL to select for stable transfectants. After stable growth was observed, concentration of geneticin was reduced to 600 µg/mL.

### NanoBRET binding assay

The cell line stably expressing the NLuc-hH_2_R construct was detached from the cell culture dishes after reaching ~ 80% confluency by treatment with trypsin/EDTA (0.05%/0.2%, for 2 min at 37 °C) and was centrifuged (600×*g*, 5 min). The cell pellet was then resuspended in Leibovitz’ L-15 medium (L-15), supplemented with 5% FCS + 10 mM HEPES, and 1.0 × 10^5^ cells/well were seeded in 70 µL (saturation and competition binding) or 80 µL (kinetic experiments) of assay medium into white 96-well cell-Grade™ plates (Brand GmbH & Co. KG, Wertheim, Germany). The cells were then incubated at 37 °C in a humidified atmosphere (no additional CO_2_) overnight. For saturation binding experiments, serial dilutions (tenfold concentrated) of the fluorescent ligands (**8**–**10**) and famotidine (**4**, 300-fold excess over the respective concentration of fluorescent ligand, non-specific binding) were prepared in dilution buffer (L-15 + 2% BSA + 10 mM HEPES). 10 µL of the fluorescent ligand dilution and 10 µL of L-15 (total binding) or the dilution of **4** (non-specific binding) were added to the cells. After 60 min incubation time at 27 °C, 10 µL of the substrate furimazine, which was diluted according to manufacturer’s protocol beforehand, were added. After 5 min of equilibration time at 27 °C, the measurement was started. Competition binding experiments were performed as described above using one fixed concentration of fluorescent ligand **8** (c = 50 nM) and varying concentrations of the competitors **1**–**4**, **11**, **12**, that were added at the same time. Kinetic measurements were performed as follows: 10 µL of L-15 (for total binding) or **4** (300-fold excess, c = 15 µM, non-specific binding) were added to the cells. After addition of the diluted substrate, the plate was placed inside the reader for 5 min to equilibrate. To start association 50 µL of a threefold concentrated solution of the fluorescent ligand **8** (c_final_ = 50 nM) were added to the adherent cells and the plate was measured for 60 min. Dissociation experiments were conducted in wells, which have been preincubated with **8** as described above for association experiments. To initiate dissociation, 50 µL of a fourfold concentrated solution of **4** (300-fold excess, c_final_ = 15 µM) were added to the cells and the measurement was performed for 4 h. All measurements were performed on a TECAN InfiniteLumi plate reader (TECAN, Grödig, Austria) at 27 °C using the Blue2 NB (460 nm ± 35 nm, bandpass) and the Red NB (> 610 nm, longpass) filter combination with an integration time of 100 ms. For the kinetic experiments, integration time was increased to 500 ms for both channels to reduce noise. BRET ratios were calculated by dividing the acceptor emission (red NB) by the donor luminescence (Blue2 NB).

For all BRET binding experiments, specific binding was calculated by subtracting non-specific binding from total binding yielding the “corrected BRET ratio”. For saturation binding experiments, total and non-specific binding were fitted simultaneously using the “one-site total and nonspecific binding” fit. Specific binding was fitted accordingly applying the “one-site specific binding” fit. For competition binding experiments, data were normalized to buffer control (0%) and a 100%-control containing solely fluorescent ligand. The normalized competition binding curves were then fitted with a four-parameter logistic fit yielding pIC_50_-values. These were transformed into p*K*_i_-values using the Cheng–Prusoff equation^38^.

For kinetic experiments, association experiments were fitted with the “one-phase association” fit yielding *k*_obs_, whereas the dissociation experiments were fitted assuming a “one-phase decay” model resulting in *k*_off_. The association rate constant *k*_on_ was calculated using the following equation: *k*_on_ = (*k*_obs_ − *k*_off_)/c(ligand); c(ligand) = 50 nM. Dissociation half-life *t*_1/2_ was calculated applying the following formula: *t*_1/2_ = ln(2)/*k*_off_.The kinetic equilibrium dissociation constant *K*_d(kin)_ was calculated as follows: *K*_d(kin)_ = *k*_off_/*k*_on_. The errors for *k*_on,_ *t*_1/2_ and *K*_d(kin)_ were calculated according to the Gaussian law of error propagation. All experimental data were analyzed using Prism 8 software (GraphPad, San Diego, CA, USA).

## Supplementary information

Supplementary Information

## Data Availability

The datasets generated and analyzed during the current study are available from the corresponding author on reasonable request.
