# Author Correction: NanoBRET binding assay for histamine H_2_ receptor ligands using live recombinant HEK293T cells

**DOI:** 10.1038/s41598-021-89954-2

**Published:** 2021-05-18

**Authors:** Lukas Grätz, Katharina Tropmann, Merlin Bresinsky, Christoph Müller, Günther Bernhardt, Steffen Pockes

**Affiliations:** Institute of Pharmacy, University of Regensburg, Universitätsstraße 31, 93053 Regensburg, Germany

Correction to: *Scientific Reports* 10.1038/s41598-020-70332-3, published online 06 August 2020.

This original version of this Article contained errors in Figures 1 and 2.

In Figure 1, in chemical structure “Cimetidine, 2”, the abbreviation N was incorrectly given as NH and its CN-group was omitted.

Additionally, in chemical structure “10, UR-KAT514”, thiophene was connected at the wrong position.

In Figure 2, in chemical structure “15, BODIPY 630/650 NHS ester” thiophene was connected at the wrong position.

The original Figures 1 and 2 and their accompanying legends appear below.

**Figure 1:**
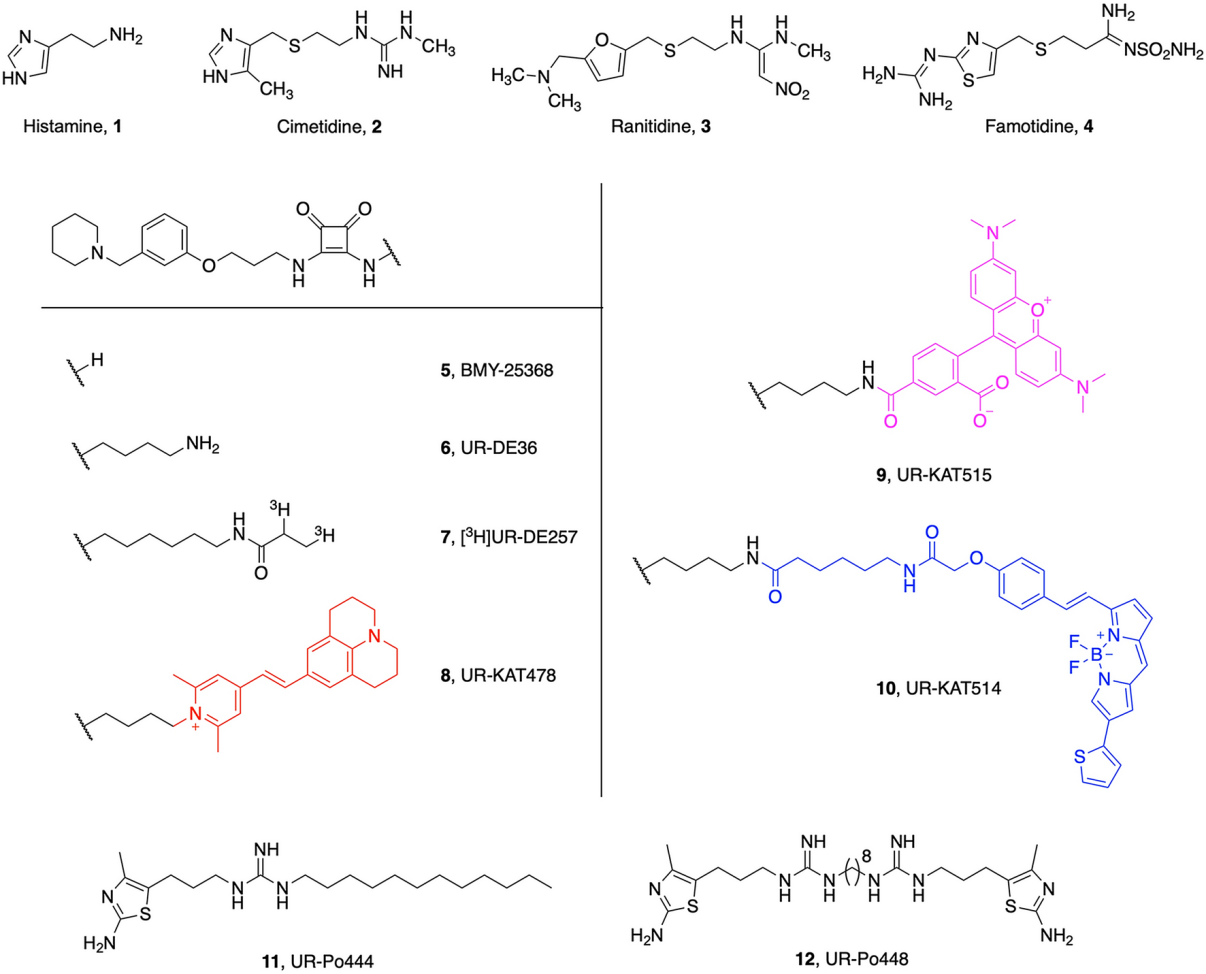
Structures of reported reference compounds (**1**–**7**, **11**–**12**) and the synthesized fluorescent ligands **8**–**10** for the histamine H_2_ receptor.

**Figure 2:**
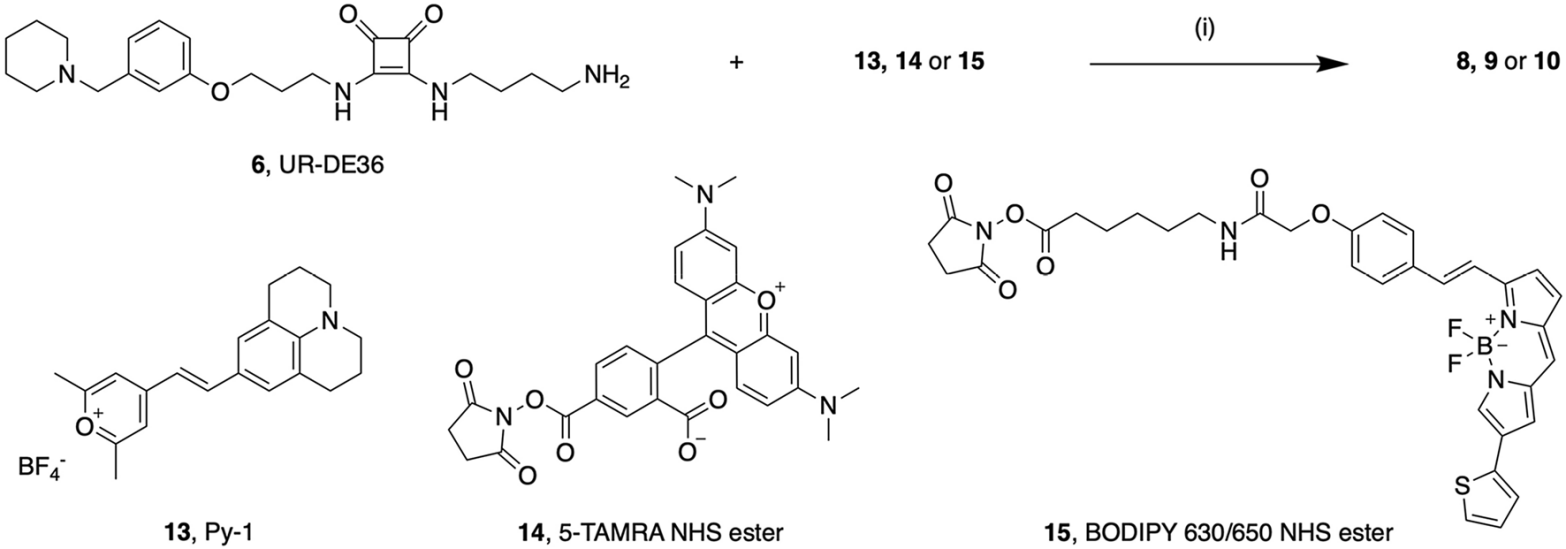
Synthesis of fluorescent ligands **8**–**10**. Reagents and conditions: (i) **6** (1.5 equiv.), NEt_3_ (7.5 or 11 equiv.), **13**, **14** or **15** (1 equiv.), DMF, rt, 2 h.

The original Article has been corrected.

