# Author Correction: NanoBRET binding assay for histamine H_2_ receptor ligands using live recombinant HEK293T cells

**DOI:** 10.1038/s41598-021-91782-3

**Published:** 2021-06-08

**Authors:** Lukas Grätz, Katharina Tropmann, Merlin Bresinsky, Christoph Müller, Günther Bernhardt, Steffen Pockes

**Affiliations:** Institute of Pharmacy, University of Regensburg, Universitätsstraße 31, 93053 Regensburg, Germany

Correction to: *Scientific Reports* https://doi.org/10.1038/s41598-020-70332-3, published online 06 August 2020

The Supplementary Information file published with this Article contained an error in Figure S6, where thiopene was connected at the wrong position.

The original Supplementary Information file is provided below.

This error has now been corrected in the Supplementary Information file that accompanies the original Article.

## Supplementary Information

Supplementary Information.

