# Supplementary material for: NanoBRET binding assay for histamine H2 receptor ligands using live recombinant HEK293T cells
Source: Sci Rep. 2020 Aug 6;10:13288. doi: 10.1038/s41598-020-70332-3 (PMC7414126; doi:10.1038/s41598-020-70332-3)
Supplement: Supplementary file 1 — Supplementary Information [file 41598_2020_70332_MOESM1_ESM.docx]

**Supplementary Information**

NanoBRET Binding Assay for Histamine H_2_ Receptor Ligands Using Live Recombinant HEK293T Cells

Lukas Grätz, Katharina Tropmann, Merlin Bresinsky, Christoph Müller, Günther Bernhardt, and Steffen Pockes*

Institute of Pharmacy, University of Regensburg, Universitätsstraße 31, D-93053 Regensburg, Germany

**Contents Pages**

1. HPLC chromatograms of 8-10 S2

2. ^1^H-NMR spectra of 8-10 S3

3. Chemical stability of fluorescent ligands 8-10 S5

4. Pharmacology S5

5. References S11

# HPLC chromatograms of 8-10

**Supplementary Figure S1.** HPLC analysis of compound UR-KAT478 (**8**, 96.0%, 220 nm).

**Supplementary Figure S2.** HPLC analysis of compound UR-KAT515 (**9**, 96.5%, 220 nm).

**Supplementary Figure S3.** HPLC analysis of compound UR-KAT514 (**10**, 98.3%, 220 nm).

# ^1^H-NMR spectra of 8-10

**Supplementary Figure S4.** ^1^H-NMR spectrum (600 MHz, DMSO-d_6_) of compound UR-KAT478 (**8**).

**Supplementary Figure S5.** ^1^H-NMR spectrum (600 MHz, DMSO-d_6_) of compound UR-KAT515 (**9**).

-

+

**Supplementary Figure S6.** ^1^H-NMR spectrum (600 MHz, DMSO-d_6_) of compound UR-KAT514 (**10**).

# Chemical stability of fluorescent ligands 8-10

The chemical stability of the fluorescent H_2_R ligands UR-KAT478 (**8**), UR-KAT515 (**9**) and UR-KAT514 (**10**) was investigated at physiological pH (7.4) in binding buffer (12.5 mM MgCl_2_, 1 mM EDTA and 75 mM Tris/HCl, pH 7.4)^1^ and in case of UR-KAT514 (**10**) as well in a mixture of DMSO/binding buffer 1:1. Incubation was started by addition of 27.5 μL of a 2.5 mM solution of the respective compounds in DMSO/Millipore H_2_O 1:1, which were freshly prepared from a 5 mM stock solution in DMSO, to 522.5 μL of binding buffer or DMSO/binding buffer 1:1 yielding a final concentration of 100 μM. The samples were shaken for up to 4 days at 700 rpm. After indicated time periods, a 70 μL aliquot was taken and diluted with 70 µL of a mixture of MeCN, Millipore H_2_O and 10% aqueous TFA (60:90:1). Prior to HPLC analysis, the samples were stored at -20 °C. 50 μL of the resulting solution were analyzed by HPLC as described in the experimental section. The absorption was detected at 220 nm. The blank HPLC run was performed under identical conditions without any ligand.

# Pharmacology

**Radioligand competition binding assay**

General procedures for the generation of recombinant baculoviruses, culture of Sf9 cells and membrane preparation are described elsewhere.^2^ Radioligand binding assays on membranes of Sf9 insect cells expressing the hH_2_R-Gsα_s_ fusion protein were performed as previously described.^1^ [^3^H]UR-DE257^3^ was used as radioligand (*K*_d_ = 11.2 nM, c = 20 nM, cf. Supplementary Fig. S7). All data analysis was performed using Prism 5.0c (GraphPad, San Diego, CA, USA).

**Supplementary Figure S7.** Representative isotherm from saturation binding with [^3^H]UR-DE257 (**7**) on membranes of Sf9 insect cells expressing the hH_2_R-Gsα_s_ fusion protein (*K*_d_ = 11.2 nM). Saturation binding assay was performed as previously described.^3^


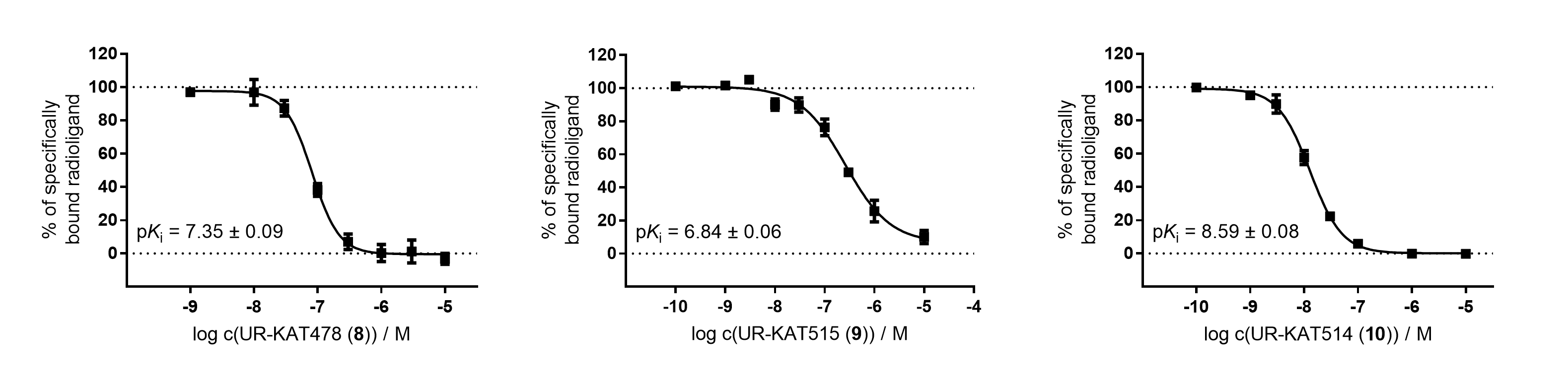


**Supplementary Figure S8.** Displacement curves of the radioligand [^3^H]UR-DE257 (*K*_d_ = 11.2 nM, c = 20 nM) with UR-KAT478 (**8**, left), UR-KAT515 (**9**, middle) and UR-KAT514 (**10**, right) performed on membrane preparations of Sf9 insect cells expressing the hH_2_R-Gsα_s_ fusion protein. Data represent mean values ± SEM from at least three independent experiments, each performed in triplicate.

**Flow cytometric saturation binding assay with HEK293T-hH_2_R-qs5-HA cells**

All flow cytometric measurements were performed with FACSCanto™II flow cytometer (Becton Dickinson, Heidelberg, Germany), equipped with an argon laser (488 nm) and a red diode laser (633 nm) (settings: FCS: A, log, 0 V; SSC: A, log, 252 V; ACP: A,H,W, log, 350 V or PerCP-Cy5-5: A,H, log 485 V or PE: A,H,W, log, 420 V) according to general protocols with minor modifications.^4,5^ All measurements were performed in duplicate and recorded either in channel PerCP-Cy5 (Py-1, excitation: 488 nm), APC (BODIPY 630/650, excitation: 633 nm) or PE (TAMRA, excitation: 488 nm). Data acquisition was stopped after 10,000 gated events.

Cell culture and preparation of HEK293T-hH_2_R-qs5-HA cells was performed as described.^4^ Briefly, cells were maintained in 25 or 175 cm^2^ flasks (Sarstedt, Nümbrecht, Germany) in a humidified atmosphere (95% air, 5% CO_2_) at 37 °C using Dulbecco`s Modified Eagle`s Medium supplemented with 2 mM L-glutamine (Sigma-Aldrich, Munich, Germany), 10% FCS, 400 µg/mL geneticin (G418), and 100 µg/mL hygromycin B (AG Scientific, San Diego, CA, USA). On the day of the experiment, cells were detached with trypsin/EDTA (0.05%/0.2%) and centrifuged (500 x *g*, 5 min). The cell pellet was resuspended in Leibovitz’ L-15 medium containing 1% FCS and the cell density was adjusted to 1 x 10^6^ cells/mL. The serial dilutions of the tested fluorescent ligands and famotidine (competitor, for non-specific binding) were prepared in DMSO/H_2_O (1:1, v/v). All incubation steps were performed in 96-well Primaria^TM^ plates (CORNING, NY, USA). 200 µL of the adjusted cell suspension were either added to 2 µL of DMSO/H_2_O (1:1, v/v, total binding) or to 2 µl of famotidine (300-fold excess over the fluorescent ligand, non-specific binding). Incubation was started by addition of 2 µL of the respective fluorescent ligand in different concentrations (100-fold concentrated with respect to the final concentration). The 96-well plate was incubated under shaking for 60 min at room temperature in the dark. Samples were transferred to 5 mL polystyrol FACS tubes (Sarstedt, Nümbrecht, Germany) and measured immediately. All data analysis was performed using Prism 5.0c (GraphPad, San Diego, CA, USA).

**Supplementary Figure S9.** Representative isotherms from flow cytometric saturation binding experiments on HEK293T-hH_2_R-qs5-HA cells with the fluorescent ligands UR-KAT478 (**8**), UR-KAT515 (**9**) and UR-KAT514 (**10**). Non-specific binding was determined in the presence of famotidine (300-fold excess). Error bars of total and non-specific binding represent SEM. Error bars of specific binding represent propagated errors calculated according to the Gaussian law of error propagation. Data shown are representative of three experiments, each performed in duplicate.

**β-Arrestin2 recruitment assay**

The recruitment of β-arrestin2 was measured employing the split-luciferase complementation technique.^6^ The luciferase complementation assay was performed with slight modifications as described by J. Felixberger using the HEK293T-ARRB2-H_2_R cell line stably expressing the H_2_R-ELucC and ELucN-ARRB2 fusion proteins.^7^ Cells were maintained in 25 or 75 cm^2^ flasks (Sarstedt, Nümbrecht, Germany) in a humidified atmosphere (95% air, 5% CO_2_) at 37 °C in Dulbecco`s Modified Eagle`s Medium supplemented with 2 mM L-glutamine, 10% FCS, 600 µg/mL G418, and 400 µg/mL zeocin (InvivoGen, Toulouse, France). One day prior to the experiment, the cells were detached with trypsin/EDTA (0.05%/0.2%) and were centrifuged (500 x *g*, 5 min). The cells were resuspended in Leibovitz’ L-15 medium + 5% FCS + 10 mM HEPES and the density of the suspension was adjusted to 1.25 x 10^6^ cells/mL (agonist mode) or 1.4 x 10^6^ cells/mL (antagonist mode). 80 µL (agonist mode) or 70 µL (antagonist mode) of the cell suspension were seeded in white, tissue culture-treated, flat bottom 96-well microtiter plates (Brand, Wertheim, Germany). The cells were incubated at 37 °C overnight in a water saturated atmosphere w/o additional CO_2_. For the determination of agonism, 10 µL of a solution of D-luciferin monopotassium salt (Thermo Scientific, Nidderau, Germany) in L-15 + 10 mM HEPES (c_final_ = 1 mM) were added per well and bioluminescence was measured for 15 min at 37 °C with an integration time of 1 s (baseline) using the EnSpire plate reader (PerkinElmer, Waltham, MA, USA). In the meantime, serial dilutions of the tested compounds (10-fold concentrated with respect to the final concentration) were prepared in L-15 + 10 mM HEPES and pre-warmed to 37 °C. After the baseline-read, the dilutions of the test compounds (10 µL/well) were transferred to the 96-well plate. After compound addition, bioluminescence was measured for 50 min at 37 °C with an integration time of 1 s. Peak luminescence values were normalized to the maximum effect induced by 1 mM histamine (100% value, positive control) and buffer control (0% value, negative control). For the determination of antagonism, the dilutions of the test compounds (10 µL/well) were transferred to the 96-well plate directly after the addition of D-luciferin (as described above) and the baseline was recorded like in the agonist mode. 10 µL of histamine (80 µM, c_final_ = 8 µM) were added per well and bioluminescence was measured for 50 min as described for the agonist mode. Resulting peak luminescence values were normalized to the effect induced by 8 µM histamine (100% value) and buffer control (0% value). Normalized data were fitted applying a four-parameter logistic fit yielding pIC_50_ values, which were then transformed into p*K*_b_ values using the Cheng-Prusoff equation.^8^ All data analysis was performed using Prism 5.0c (GraphPad, San Diego, CA, USA).

**Supplementary Figure S10.** β-Arrestin2 recruitment assay (antagonist mode) with compounds UR-KAT478 (**8**), UR-KAT515 (**9**) and UR-KAT514 (**10**). The graphs display the inhibition of the histamine-induced (EC_50_ = 3.8 µM, c = 8 µM) response (β-arrestin2 recruitment) in HEK293T-ARRB2-hH_2_R cells. Data points represent the mean values ± SEM from at least three independent experiments, each performed in triplicate.


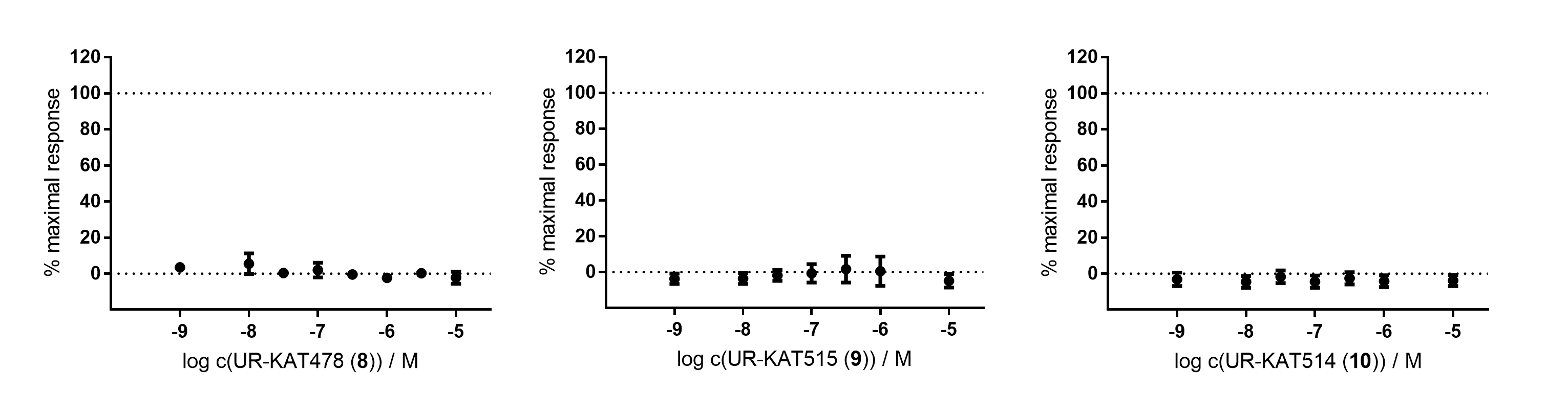


**Supplementary Figure S11.** β-Arrestin2 recruitment assay (agonist mode) with compounds UR-KAT478 (**8**), UR-KAT515, (**9**) and UR-KAT514 (**10**). The graphs display compound-induced β-arrestin2 recruitment in HEK293T-ARRB2-hH_2_R cells. Data points represent the mean values ± SEM from at least three independent experiments performed in triplicate. The four-parameter logistic fits of the data from individual experiments failed.

**Supplementary Figure S12.** Competition binding experiment (N = 3, each performed in triplicate) with histamine and **8** (c = 50 nM) at HEK293T cells, stably expressing the NLuc-H_2_R. A representative experiment with monophasic (left panel, black) and biphasic (right panel, red) fit is presented. Error bars represent SEM. Data shown are representative of three experiments, each performed in triplicate.

# References

(1) Kagermeier, N.; Werner, K.; Keller, M.; Baumeister, P.; Bernhardt, G.; Seifert, R.; Buschauer, A. Dimeric Carbamoylguanidine-Type Histamine H2 Receptor Ligands: A New Class of Potent and Selective Agonists. *Bioorg. Med. Chem.* **2015**, *23* (14), 3957–3969. https://doi.org/10.1016/j.bmc.2015.01.012.

(2) Pop, N.; Igel, P.; Brennauer, A.; Cabrele, C.; Bernhardt, G.; Seifert, R.; Buschauer, A. Functional Reconstitution of Human Neuropeptide Y (NPY) Y2 and Y4 Receptors in Sf9 Insect Cells. *J. Recept. Signal Transduct.* **2011**, *31* (4), 271–285.

(3) Baumeister, P.; Erdmann, D.; Biselli, S.; Kagermeier, N.; Elz, S.; Bernhardt, G.; Buschauer, A. [3H]UR-DE257: Development of a Tritium-Labeled Squaramide-Type Selective Histamine H2 Receptor Antagonist. *ChemMedChem* **2015**, *10* (1), 83–93. https://doi.org/10.1002/cmdc.201402344.

(4) Mosandl, J. Radiochemical and Luminescence-Based Binding and Functional Assays for Human Histamine Receptors Using Genetically Engineered Cells. PhD Thesis, University of Regensburg, 2009.

(5) Schneider, E.; Mayer, M.; Ziemek, R.; Li, L.; Hutzler, C.; Bernhardt, G.; Buschauer, A. A Simple and Powerful Flow Cytometric Method for the Simultaneous Determination of Multiple Parameters at G Protein-Coupled Receptor Subtypes. *ChemBioChem* **2006**, *7* (9), 1400–1409. https://doi.org/10.1002/cbic.200600163.

(6) Lieb, S.; Littmann, T.; Plank, N.; Felixberger, J.; Tanaka, M.; Schäfer, T.; Krief, S.; Elz, S.; Friedland, K.; Bernhardt, G. Label-Free versus Conventional Cellular Assays: Functional Investigations on the Human Histamine H1 Receptor. *Pharmacol. Res.* **2016**, *114*, 13–26.

(7) Felixberger, J. Luciferase Complementation for the Determination of Arrestin Recruitment: Investigations at Histamine and NPY Receptors. PhD Thesis, University of Regensburg, 2014.

(8) Cheng, Y.-C.; Prusoff, W. H. Relationship between the Inhibition Constant (K_i_) and the Concentration of the Inhibitor Which Causes 50 per Cent Inhibition (I_50_) of an Enzymatic Reaction. *Biochem. Pharmacol.* **1973**, *22*, 3099–3108.
